# The Present State of Lunacy in England and Wales

**Published:** 1858-10-01

**Authors:** 


### Art. VI.—THE PRESENT STATE OF LUNACY IN ENGLAND AND WALES.

The Twelfth Report of the Commissioners in Lunacy has been recently published, and it contains much important information respecting the public and private provision for lunatics, and the statistics of lunacy in England and Wales. The Report is dated the 31st of March this year, and it would appear that steps have been taken in several counties to provide increased accommodation for pauper lunatics, and that considerable progress has in this respect been made since the previous Report. New sites have been purchased, and plans for additional asylums have been prepared, and have received the approval of the Secretary of State; buildings already in course of erection have been materially advanced towards completion; and additions have been made, or commenced, at several existing asylums where the accommodation had been found inadequate to the wants of the district.

The present accommodation for pauper lunatics in existing asylums in England and Wales, amounts to 7516 beds (including infirmary beds) for males, and 8715 beds for females. The Report states that, when—

"The buildings now in progress are completed, the total number of additional beds will be as follows:

| | |
|---|---|
| In new Asylums . . . . . . . . . | 2336 |
| Additions to existing Asylums . . . . | 2481 |
| Total . . . . . . | 4817 |

And if to this estimate of the most recent additions to the public accommodation provided for pauper lunatics, we apply the ratio of increase in the numbers requiring accommodation observable during the last year, some conclusion may be formed as to the period for which these additional beds are likely to be found sufficient to meet the constantly increasing wants of the country, and how far they will tend towards the object we have sought most anxiously to promote ever since the establishment of this commission, namely, the ultimate closing of licensed houses for pauper patients.

"On the 1st of January, 1857, the number of pauper lunatics in the county and borough asylums, hospitals, and licensed houses amounted to 16,657. On the 1st of January, 1858, this number had increased to 17,572, showing an increase during the year of 915 patients. And of the total number, 2467 were confined in the various metropolitan and provincial licensed houses.

Assuming, then, that during the next two years the progressive increase in the number of pauper lunatics will be at least equal to that of the year 1857, it follows that on the 1st of January, 1860, accommodation for 1830 additional patients will be required; and if to this number be added the 2467 patients who are now confined in licensed houses, there will remain to meet the wants of the ensuing year only 520 vacant beds. It is obvious, therefore, that if licensed houses are to be closed for the reception of pauper patients, some scheme of a far more comprehensive nature must be adopted in order to provide public accommodation for the pauper lunatics of this country."

The following table shows the situation and projected accommodation of the asylums now in contemplation or in course of erection, and the period when they will probably be fit for occupation:—

| — | Number of patients for whom accommodation will be provided. | | When to be completed. | — | Number of patients for whom accommodation will be provided. | | When to be completed. |
|---|---|---|---|---|---|---|---|
| | M. | F. | | | M. | F. | |
| Cumberland | 100 | 100 | October, 1860. | Sussex . . . . | 200 | 200 | Mar. 1, 1859. |
| Durham . . | 157 | 155 | December 1, 1858. | Cambridge . . | 125 | 125 | May, 1858. |
| Carmarthen. | 135 | 135 | Quite uncertain. | Beds, Herts, and Hunts . . . | 252 | 252 | Uncertain. |
| Bristol . . | 100 | 100 | 1859. | Northumberland | 100 | 100 | Sept. 1, 1858. |

The aggregate cost of these new asylums will amount to 381,827*l*.

The Commissioners objected to the northerly slope of the land upon which the Bristol Asylum is to be built, also to the insufficiency of the ground in quantity proportionately to the number of patients to be accommodated, and the site was approved of only after the assurance of the Committee appointed by the Corporation of the City to carry out the provisions of the Act, that it was impossible to procure a larger quantity of land at a reasonable price, within a convenient distance of Bristol. The Commissioners objected also to the extent of the site selected for the Carmarthen, Cardigan, and Pembroke Asylum. The space they considered was not sufficiently large for the number of patients to be provided for, and they remark—"Our experience has satisfied us that in no case should the land belonging to an asylum be less than in the proportion of one acre to four patients." The Committee of Visitors did not concur with the opinion of the Commissioners, and the purchase of the site was authorized by the Secretary of State, as there were pecuniary difficulties in the way of obtaining another.

During the erection of a new building for the accommodation of female patients at the Devon County Asylum, the experiment was tried of removing a number of female patients from the asylum to a hired house at Exmouth, pending the erection of the building. The Commissioners report that this experiment "has proved entirely successful," and they add—

"Forty-two patients together have generally been accommodated, the individuals being changed from time to time; and the house was visited in the month of September by two members of our Board, who reported very favourably of the condition of the inmates. They found them well attended to, and comfortable in every respect. The alarm which had originally existed in the minds of the inhabitant of Exmouth, when it was first proposed to place the patients there, appeared to have entirely subsided. Although the majority of the patients were daily taken out in parties for walks by the shore, and in the neighbourhood, no inconvenience had in any case been felt; no individual was annoyed in any way; and, after a very short period, the patients themselves ceased to attract any unusual attention, being indeed soon considered as forming a part of the ordinary inhabitants of the town.

"It is to be hoped that the committees of other asylums may be induced to follow the example thus set by the Devonshire justices, now that so convincing a proof has been afforded of its practicability and success."

The Commissioners report upon the defective management of the Haverfordwest Asylum. It would seem that the hygienic condition of the buildings and the patients was highly and unnecessarily imperfect, and that measures of cruel restraint were from time to time had recourse to, no record of such measures being entered in the medical journal. Notwithstanding repeated remonstrances of the Commissioners, no improvement had taken place in the management of the asylum at their last visit in September, 1857, and in consequence, upon their recommendation, the medical officer was superseded. Several acts of cruelty to patients in one of the refractory wards of the Northampton Hospital for the Insane were also ascertained to have been committed by three of the attendants, upon an investigation of certain charges which had been brought to the knowledge of the Commissioners. The offenders were forthwith dismissed, the Commissioners having expressed the opinion that such a step was imperatively necessary.

It would seem that there is now an immediate prospect of a suitable site being obtained, and of the erection of an asylum without delay, for the City of London, the Aldermen having come to an arrangement with the Common Council in reference to questions of funds and rate.

In commenting upon the proposed enlargements of the Hanwell and Colney Hatch Asylums (in the former of which asylums additional accommodation is to be provided for 348 males and 362 females, in the latter for 365 males and 431 females), the Commissioners express their disapprobation of "the enlargement of these already overgrown establishments," and they remark upon the plans according to which the enlargement of the Colney Hatch Asylum is now being carried out, that,

"The most important suggestion made by us in reference to these plans was, that instead of so proposing to add new wards, constructed upon the very extensive design carried out in the original asylum, a distinct and separate building should be erected for the reception of quiet, convalescent, and working patients to the number of about 600, leaving only an addition of 200 to be accommodated in the main building. But to this proposition the Committee of Visitors declined to accede; and after a considerable amount of correspondence, the original plans were proceeded with, subject to various minor alterations and modifications in matters of detail suggested by ourselves and our consulting architect. In stating these circumstances, we are bound to add that all our experience only tends to strengthen the opinions we have formerly expressed upon this subject; and we feel well assured that, had our propositions been adopted, not only would the ratepayers of the county have been spared a very large outlay, but the patients themselves would have derived benefit from being lodged in rooms more cheerful, and better adapted to their previous habits, than the monotonous wards and galleries of the asylum. We think it right, therefore, as in the case of Hanwell, here to place on record the regret we feel that the Committee of Visitors of the Colney Hatch Asylum should have lost so favourable an opportunity of making efficient provision for a large portion of the pauper lunatics of the county of Middlesex, at a cost so much below what will be (and has hitherto been) incurred for this purpose."

In reference to the existing *Hospitals for the Insane*, the Commissioners express the opinion that the system of part-payment by the friends of the patient might be advantageously adopted to a greater extent than is at present the case, more especially in the Hospitals of St. Luke and Bethlehem; and they also state their regret that the Governors of Guy's Hospital should have decided to receive no more patients into the lunatic wards of that hospital, and should have come to the determination to close the wards altogether when the present inmates shall have died.

In the last Report of the Commissioners it was stated that the Government had determined to provide a new State Asylum for Criminal Lunatics. In pursuance of that determination 290 acres of land have been purchased, at the moderate cost of 6000*l.*, on Bagshot Heath, and the Commissioners report that the site is well fitted for the object to which it will be devoted.

The condition of *single patients* has engaged much of the Commissioners' attention during the past year, and they have been led to form an unfavourable opinion of the management and accommodation of these patients. "The lists have been carefully revised; regular returns from the persons having charge of patients have been strictly enforced; and the country has been divided into districts so as to insure the regular annual visitation of all cases returned to our office;" but the Commissioners state, that,

"On the whole, the condition of the patients so visited cannot be described as satisfactory. As a general rule, the accommodation provided is quite incommensurate with the payments, which in many instances are very large. The necessity for our continued and regular supervision has been clearly established, and in some instances we have found cases of marked neglect. In two or three we have discovered that, besides the patient who has been regularly certified and returned to this office, the proprietors of houses have also had under their charge other persons of unsound mind, relative to whom no return whatever had been made. In these cases, although, from the presence of circumstances of an extenuating character, we have not deemed it proper to institute prosecutions for the legal penalties incurred, we have insisted, by way of warning to others against commission of the same or any similar offence, that the offenders should insert apologies in the daily and medical papers. In the majority of cases we have found that the provisions of the law as to the visitations by a second medical man, the keeping of a medical journal, and the annual and other returns to be made to our office, have been totally or partially neglected; and in every instance we have taken such steps as were necessary to secure in future a more strict compliance with the provisions of the law. Our experience on this head during the past year, in short, has confirmed the impression we have long entertained, that a very large number of insane persons are taken charge of by medical men and others without any legal authority; and, judging from the cases which have come to our knowledge, we have reason to fear that the condition of such patients, deprived as they are of all independent supervision, is far from satisfactory."

The Commissioners suggest an amendment in the Lunatic Asylums Act of 1853, in order to remove certain difficulties which exist in the provision of accommodation for pauper lunatics in boroughs; and the Report terminates with an Appendix containing several tables, from which the following statistics of lunacy are obtained:—

*Return of Insane Persons confined in Asylums, Hospitals, and Licensed Houses on the 1st January, 1858.—Summary.*

| — | Private. | | | Pauper. | | | Total Males. | Total Females. | Total Lunatics. |
|---|---|---|---|---|---|---|---|---|---|
| | M. | F. | Total. | M. | F. | Total. | | | |
| Asylums . . . . . | 134 | 98 | 232 | 6,797 | 8,134 | 14,931 | 6,931 | 8,232 | 15,163 |
| Hospitals . . . . . | 818 | 759 | 1,577 | 95 | 79 | 174 | 913 | 838 | 1,751 |
| Metropolitan Licensed Houses . . . . . | 676 | 630 | 1,306 | 490 | 827 | 1,317 | 1,166 | 1,457 | 2,623 |
| Provincial Licensed Houses . . . . . | 754 | 743 | 1,497 | 603 | 547 | 1,150 | 1,357 | 1,290 | 2,647 |
| | 2,382 | 2,230 | 4,612 | 7,985 | 9,587 | 17,572 | 10,367 | 11,817 | 22,184 |
| Royal Naval Hospital . | 126 | — | 126 | — | — | — | 126 | — | 126 |
| | 2,508 | 2,230 | 4,738 | 7,985 | 9,587 | 17,572 | 10,493 | 11,817 | 22,310 |

| — | Found lunatic by inquisition. | | | Criminals. | | | Chargeable to Counties or Boroughs. | | |
|---|---|---|---|---|---|---|---|---|---|
| | M. | F. | Total. | M. | F. | Total. | M. | F. | Total. |
| Asylums . . . . . | 10 | 2 | 12 | 224 | 79 | 303 | 590 | 664 | 1,254 |
| Hospitals . . . . . | 18 | 13 | 31 | 98 | 20 | 118 | — | — | — |
| Metropolitan Licensed Houses . . . . . | 64 | 58 | 122 | 29 | 6 | 35 | 47 | 56 | 103 |
| Provincial Licensed Houses . . . . . | 79 | 51 | 130 | 139 | 38 | 177 | 57 | 76 | 133 |
| | 171 | 124 | 295 | 490 | 143 | 633 | 694 | 796 | 1,490 |
| Royal Naval Hospital . | — | — | — | — | — | — | — | — | — |
| | 171 | 124 | 295 | 490 | 143 | 633 | 694 | 796 | 1,490 |

*Return of Admissions, Discharges, and Deaths of Patients of all Classes in Asylums, Hospitals, and Licensed Houses, during the year* 1857. *—Summary.*

| — | No. of Patients 1st Jan., 1857. | | Admissions during 1857. | | Discharges during 1857. Total number. | | Discharges during 1857. Number recovered. | | Deaths during 1857. Total number. | | Deaths during 1857. From suicide. In the asylum. | | Deaths during 1857. From accident or violence. In the asylum. | | Patients remaining 1st Jan., 1858. Total number. | | Patients remaining 1st Jan., 1858. Number deemed curable. | |
|---|---|---|---|---|---|---|---|---|---|---|---|---|---|---|---|---|---|---|
| | M. | F. | M. | F. | M. | F. | M. | F. | M. | F. | M. | F. | M. | F. | M. | F. | M. | F. |
| County and Borough Asylums | 6570 | 7825 | 2384 | 2397 | 1149 | 1340 | 843 | 1011 | 871 | 667 | 5 | 2 | 6 | 3 | 6914 | 8211 | 839 | 990 |
| Hospitals | 907 | 826 | 382 | 408 | 308 | 336 | 146 | 193 | 65 | 47 | — | — | 1 | — | 912 | 839 | 82 | 116 |
| Metropolitan Licensed Houses | 1125 | 1451 | 523 | 548 | 326 | 414 | 125 | 173 | 160 | 116 | 2 | — | — | — | 1167 | 1456 | 135 | 190 |
| Provincial Licensed Houses | 1360 | 1173 | 606 | 647 | 496 | 468 | 185 | 189 | 136 | 84 | 4 | 1 | 2 | — | 1353 | 1280 | 199 | 232 |

